# Successful Laparoscopic Sleeve Gastrectomy in a Patient With Rare Congenital Factor VII Deficiency: A Case Report and Management Strategy

**DOI:** 10.7759/cureus.75903

**Published:** 2024-12-17

**Authors:** Khalid M Alzahrani, Abdulaziz H Basha Ahmed, Haroun A Almoumani, Abdallah Elzeiny, Hafiz A Hamdi

**Affiliations:** 1 Department of Surgery, College of Medicine, Taif University, Taif, SAU; 2 Department of Medicine, Al-Noor Specialist Hospital, Makkah, SAU; 3 Department of General and Bariatric Surgery, Saudi German Hospital, Makkah, SAU; 4 Department of Surgery, King Abdulaziz Specialist Hospital, Taif, SAU

**Keywords:** coagulopathy, congenital fvii deficiency, laparoscopic sleeve gastrectomy, metabolic bariatric surgery, surgical bleeding

## Abstract

Congenital factor VII (FVII) deficiency is a rare coagulation disorder that increases the risk of bleeding complications during surgery. Although laparoscopic sleeve gastrectomy (LSG) is the most common metabolic bariatric surgery (MBS), it is rarely performed in patients with congenital coagulation disorders such as FVII deficiency, due to the high risk of intraoperative and postoperative bleeding. We report the case of a 57-year-old female with class II obesity (BMI 37.9 kg/m²) and previously undiagnosed congenital FVII deficiency presenting for LSG. The patient’s prothrombin time-international normalized ratio (PT-INR) was elevated (2.3; normal range: 0.8-1.2) with a normal activated partial thromboplastin time (aPTT) and no known preexisting liver disease or use of anticoagulant medications. Further hematology workup confirmed isolated low FVII activity (11% of normal). Preoperative management included transfusion of two units of fresh frozen plasma (FFP) to correct the coagulopathy. Intraoperatively, 1000 international units (IU) of prothrombin complex concentrate (PCC) were administered before the end of surgery. The procedure proceeded uneventfully with no bleeding complications. Postoperatively, thromboprophylaxis with low molecular weight heparin (LMWH) was administered for 14 days without any bleeding episodes. This case demonstrates that with careful preoperative evaluation, appropriate correction of coagulopathy, and a multidisciplinary team approach, LSG can be safely performed in patients with congenital FVII deficiency. This report may serve as a reference for managing similar high-risk patients undergoing major surgeries.

## Introduction

Congenital factor VII (FVII) deficiency is a rare autosomal recessive coagulation disorder, affecting approximately one in 500,000 individuals worldwide and ranking among the least common inherited coagulopathies [1]. FVII, a vitamin K-dependent protein synthesized in the liver, is crucial for initiating the extrinsic coagulation pathway by binding to tissue factors and thereby activating downstream clotting factors [2]. Deficiency of FVII manifests in a spectrum of clinical presentations, ranging from asymptomatic individuals to those with severe bleeding episodes. The International Society on Thrombosis and Haemostasis Scientific Standardization Committee (ISTH-SSC) classifies FVII deficiency based on FVII activity levels: severe deficiency (<10%) is associated with spontaneous major bleeding, moderate deficiency (10-20%) often presents with mild spontaneous or trauma-induced bleeding, and mild deficiency (20-50%) may remain largely asymptomatic [3].

Diagnosis of FVII deficiency is often prompted by an isolated prolongation of prothrombin time-international normalized ratio (PT-INR) with a normal activated partial thromboplastin time (aPTT), followed by specific factor assays to confirm the disorder [4]. Although substitution therapy with fresh frozen plasma (FFP), prothrombin complex concentrate (PCC), or recombinant activated FVII (rFVIIa) is widely accepted for patients undergoing surgery with severely low FVII levels or a history of recurrent bleeding, standardized guidelines are lacking due to the rarity of the condition [1,5]. This scarcity of data poses considerable challenges, especially in major surgical procedures where bleeding risks are inherently high.

Laparoscopic sleeve gastrectomy (LSG) is a commonly performed metabolic bariatric surgery (MBS), widely accepted for treating obesity and related comorbidities. The procedure’s minimally invasive nature, leading to potentially reduced blood loss and fewer postoperative complications, makes it appealing for patients at risk of bleeding [6]. However, performing LSG in patients with congenital FVII deficiency is rarely reported, and there is limited guidance on how best to manage these patients perioperatively.

In this case report, we present the successful management of a patient with congenital FVII deficiency undergoing LSG. By detailing the perioperative interventions, ranging from careful preoperative evaluation and factor correction to intraoperative hemostatic support and thoughtful postoperative care, this report aims to fill a gap in the literature. Such evidence can guide clinicians facing similar challenges, ultimately improving outcomes for patients with rare bleeding disorders undergoing major surgeries [2-6].

## Case presentation

A 57-year-old female patient with class II obesity (BMI 37.9 kg/m²) and hypertension presented to the bariatric surgery clinic seeking MBS. She had a past medical history significant for a major bleeding episode following a cesarean section 20 years earlier, but no formal diagnosis of coagulopathy had ever been established. She reported no current use of anticoagulant medications and no known liver disease.

Preoperative laboratory evaluation revealed a PT-INR of 2.3 (normal range: 0.8-1.2), with a normal aPTT of 29 seconds and a normal platelet count of 253,000/µL. Comprehensive blood counts, thyroid function tests, serology (hepatitis C virus, hepatitis B surface antigen, and human immunodeficiency virus), and liver function tests were all normal. The isolated elevation in PT-INR with normal aPTT prompted a hematology consultation, and coagulation factor assays confirmed isolated low FVII activity at 11% of normal, establishing the diagnosis of congenital FVII deficiency.

Preoperative optimization included the transfusion of two units of FFP to replenish factor FVII. Under general anesthesia, LSG was performed laparoscopically. The patient was positioned in the reverse Trendelenburg position with a split-leg supine configuration, and the surgeon stood between the patient’s legs. Three laparoscopic ports were placed: a 10-mm optical trocar for the camera, a 12-mm trocar in the left upper quadrant, and a 5-mm trocar approximately 4 cm below the left subcostal anterior axillary line. The greater curvature of the stomach was mobilized by dividing the gastrocolic ligament with a LigaSure™ device (5 mm-37 cm) (Medtronic Plc, Dublin, Ireland). A 36 French bougie was inserted along the lesser curvature to guide the gastric resection. The stomach was divided using an Endo GIA™ ultra universal stapler (Medtronic Plc) with four purple and one gold 60 mm staple loads, beginning approximately 4 cm from the pylorus and proceeding to the angle of His. Meticulous attention was paid to ensuring hemostasis and preventing bleeding. The resected portion of the stomach was extracted through one of the port sites. A leak test with 50 ml of methylene blue was performed to confirm the integrity of the staple line. The procedure was completed without intraoperative complications or bleeding episodes. To ensure adequate hemostatic support during the critical operative period, 1000 IU of PCC was administered before the conclusion of the surgery. The procedure was completed uneventfully, and no intraoperative bleeding complications were observed.

Postoperatively, the patient received low molecular weight heparin (LMWH) thromboprophylaxis for 14 days to mitigate the increased thrombotic risk associated with obesity and surgery. She experienced no bleeding episodes throughout her hospitalization and was discharged without complications, demonstrating a successful outcome.

Postoperative follow-up at two weeks and three, six, nine, and 12 months showed normal recovery with no active complaints. At the 18-month follow-up, the patient’s BMI had decreased to 23.5, and she had no active complaints.

## Discussion

Managing patients with congenital FVII deficiency undergoing major surgical interventions remains a significant clinical challenge due to the high risk of bleeding and the lack of standardized management protocols [1-2]. Prompt diagnosis and early intervention are essential. In this case, an isolated elevation of PT-INR with a normal aPTT provided a strong clue toward a clotting factor deficiency. Subsequent factor assays confirming low FVII activity guided the perioperative management strategy [4].

Preoperative management

The preoperative strategy focused on optimizing hemostasis to mitigate bleeding risks during surgery. FFP administration was used to provide multiple clotting factors, including FVII, effectively bridging the patient through the perioperative period when bleeding risk is heightened [5]. Intraoperative administration of PCC further ensured adequate FVII levels during surgery. While rFVIIa is often considered the ideal therapy for addressing FVII deficiency directly, factors such as institutional availability, cost constraints, and established protocols influenced the selection of hemostatic agents in this case [2-3,6].

The preoperative preparation, including the correction of clotting abnormalities, was instrumental in preventing intraoperative bleeding, particularly as the patient underwent an LSG.

Intraoperative strategy

The laparoscopic approach was chosen to minimize the risk of hemorrhagic complications, which are particularly concerning in patients with bleeding disorders. Minimally invasive techniques are associated with reduced blood loss compared to open surgical methods, making them a preferred option for such high-risk cases [6]. This decision likely contributed to the uneventful surgical course, as no intraoperative bleeding was observed, despite the underlying coagulopathy. The administration of PCC ensured that FVII levels remained adequate throughout the surgery, further reducing the risk of bleeding complications.

Postoperative outcomes

Postoperative management in patients with congenital FVII deficiency undergoing bariatric surgery involves a careful balance between preventing thromboembolic events and avoiding bleeding complications. This balance is particularly complex due to the hypercoagulable state associated with obesity and surgery. Prophylactic anticoagulation with low LMWH was initiated to prevent venous thromboembolism (VTE). LMWH was selected due to its favorable pharmacokinetics, predictable anticoagulant effect, and lower risk of complications, such as heparin-induced thrombocytopenia (HIT).

In this case, LMWH was administered at a prophylactic dose 24 hours postoperatively to ensure hemostatic stability and reduce the risk of VTE without provoking bleeding. The patient remained closely monitored for any signs of bleeding or thrombotic complications, allowing for prompt intervention if needed. The absence of both thrombotic and bleeding events underscores the effectiveness of a tailored, multidisciplinary approach to postoperative care in this unique patient population [5,6].

This report contributes to the limited literature on performing LSG in patients with congenital FVII deficiency. Detailing a successful perioperative strategy, it highlights the importance of individualized management plans driven by collaboration among surgeons, hematologists, anesthesiologists, and nursing staff. As additional cases are reported and data accumulate, the development of clearer guidelines will enhance the safety and efficacy of surgical interventions in this rare patient population.

## Conclusions

This case demonstrates the successful performance of LSG in a patient with congenital FVII deficiency through a comprehensive perioperative approach that included early recognition, appropriate factor replacement, meticulous surgical technique, and careful postoperative monitoring. The favorable outcome underscores the potential for safe and effective major surgery in patients with rare bleeding disorders when guided by a multidisciplinary strategy and individualized treatment plan. As the literature on this topic remains sparse, this experience may serve as a valuable reference, informing clinicians faced with similar challenges and contributing to the gradual development of evidence-based guidelines for managing congenital FVII deficiency in major surgical settings .
